# Ablation of C/EBP Homologous Protein Does Not Protect T17M *RHO* Mice from Retinal Degeneration

**DOI:** 10.1371/journal.pone.0063205

**Published:** 2013-04-30

**Authors:** Sonali Nashine, Yogesh Bhootada, Alfred S. Lewin, Marina Gorbatyuk

**Affiliations:** 1 Department of Cell Biology and Anatomy, University of North Texas Health Science Center, Fort Worth, Texas, United States of America; 2 Department of Vision Sciences, University of Alabama at Birmingham, Birmingham, Alabama, United States of America; 3 Department of Molecular Genetics and Microbiology, University of Florida, Gainesville, Florida, United States of America; University of Cologne, Germany

## Abstract

Despite the proposed link between ablation of the CHOP protein and delay of the onset of ER stress-mediated disorders including diabetes, Alzheimer Disease, and cardiac hypertrophy, the role of CHOP protein in photoreceptor cell death associated with Autosomal Dominant Retinitis Pigmentosa (ADRP) has not been investigated. T17M *RHO* transgenic mice carry a mutated human rhodopsin transgene, the expression of which in retina leads to protein misfolding, activation of UPR and progressive retinal degeneration. The purpose of this study is to investigate the role of CHOP protein in T17M *RHO* retina. Wild-type, CHOP−/−, T17M *RHO* and T17M *RHO* CHOP−/−mice were used in the study. Evaluation of the impact of CHOP ablation was performed using electroretinography (ERG), spectral-domain optical coherence tomography (SD-OCT), quantitative Real-Time PCR (qRT-PCR) and western blot analysis. Dark-adapted ERG analysis demonstrated that by 1 month, the T17M *RHO* CHOP−/− mice had a 70% reduction of the a-wave amplitude compared to the T17M *RHO* mice. The loss of function in T17M *RHO* CHOP−/− photoreceptors was associated with a 22–24% decline in the thickness of the outer nuclear layer. These mice had significant reduction in the expression of transcription factors, *Crx* and *Nrl*, and also in mouse *Rho*, and human *RHO.* The reduction was associated with an 8-fold elevation of the UPR marker, p-eIf2α protein and 30% down-regulation of sXbp1 protein. In addition, the histone deacetylase 1 (Hdac1) protein was 2-fold elevated in the T17M *RHO* CHOP−/− retina. The ablation of CHOP led to a reduction in the expression of photoreceptor-specific transcriptional factors, and both endogenous and exogenous *RHO* mRNA. Thus, despite its role in promoting apoptosis, CHOP protects rod photoreceptors carrying an ADRP mutation.

## Introduction

Autosomal dominant forms of progressive inherited retinal degeneration, retinitis pigmentosa (RP) account for approximately 30% of all RP cases [1]. Mutations in rhodopsin (RHO) are the most prevalent class identified to date, causing 25% of all ADRP cases [1]. The clinical manifestation of RP includes loss of sensitivity to dim light, abnormal visual function, and characteristic bone spicule deposits of pigment in the retina [2]. Affected individuals progressively lose their visual field and visual acuity, and photoreceptor cell death can ultimately lead to blindness [2]. An AGT or ACG subsititution in codon 17 of *RHO* leads to the replacement of the amino acid, threonine with methionine, possibly affecting glycosylation at asparagine 19 [3]. T17M is considered a Class II *RHO* mutation because it is characterized by the inability of mutant opsin to form functional rhodopsin with 11-cis-retinal and by opsin accumulation in the endoplasmic reticulum (ER) and Golgi apparatus. The accumulation of these mutant misfolded proteins in the ER triggers a signal transduction cascade known as the Unfolded protein Response (UPR) [4], resulting in the activation of c-Jun and apoptosis [5].

The CHOP (C/EBP Homologous Protein, also known as GADD153 and DDIT3) gene encodes a member of the CCAAT/enhancer-binding protein (C/EBP) family of transcription factors. CHOP is a 29 kDa protein consisting of 169 (human) or 168 (rodent) amino acid residues [6]. The protein functions as a dominant-negative inhibitor by forming heterodimers with other C/EBP members, such as C/EBP and LAP (liver activator protein), and blocking their DNA binding activity [6]. It plays an important role in ER stress-induced apoptosis. CHOP induces apoptosis via dephosphorylation of phosphorylated (p) eukaryotic translation initiation factor 2 alpha (eIF2α), down-regulation of the expression of the anti-apoptotic protein Bcl-2, and translocation of the pro-apoptotic molecule Bax from the cytosol to the mitochondria [6]. It is ubiquitously expressed at very low levels. However, its expression increases several fold under conditions of stress in a wide variety of cells. CHOP is present in the cytosol under non-stressed conditions, and stress leads to the induction of CHOP and its accumulation in the nucleus.

It has been shown that CHOP is involved in macrophage apoptosis induced by combinations of ER stressors and pattern recognition receptor ligands [7], [8], and deletion of CHOP blocks apoptosis without leading to default necrosis [7]. Previous studies have also demonstrated that disruption of the CHOP gene protects the islet cells of Ins2^WT/C96Y^ mice from apoptosis, thus delaying the onset of ER stress-mediated diabetes [9] **.** Recent findings also indicate that ER stress-mediated CHOP activation plays a central role in causing Alzheimer Disease (AD) pathology by leading to cholesterol oxidization to produce the metabolite 27-hydroxycholesterol (27-OHC) [10]. However, decreasing CHOP protein leads to reduction of β-amyloid precursor protein (APP) and β-secretase (BACE1), which cleaves α-β peptide, suggesting that preventing Gadd153 activation protects against AD symptoms related to oxidized cholesterol products [10]. In CHOP-deficient murine models of atherosclerosis, such as fat-fed Chop+/+;Apoe−/− and Chop−/−;Apoe−/− mice, as well as fat-fed Chop−/−;Ldlr−/− versus Chop+/+;Ldlr−/− mice, reductions in lesion area due to substantial reduction of plaque necrosis and intimal apoptosis have been associated with deficits in CHOP protein [11]. In addition, it has been discovered that mice lacking CHOP show less cardiac hypertrophy, fibrosis, and cardiac dysfunction compared with wild-type mice after transverse aortic constriction induced by pressure overload [12]. In the hearts of CHOP-deficient mice, phosphorylation of eIf2α, which may reduce protein translation, is enhanced compared to that of wild-type mice. The last study also proposed the novel concept that CHOP, which may modify protein translation and mediate ER-initiated apoptotic cell death, contributes to the development of cardiac hypertrophy and failure, leading to myocyte apoptosis [12].

Despite the large body of literature linking CHOP protein to many disease conditions, there have been no studies correlating the role of CHOP and photoreceptor cell death associated with retinitis pigmentosa or any of the retinal degenerative diseases. However, in our laboratory, we have demonstrated that there is an increase in CHOP protein production and gene expression as a response to ER stress related to ADRP in different rodent models, including T17M *RHO* mice [4], [13], [14]. The relationship between CHOP protein and RHO, under the influence of ER stress has been recently described in HEK293T and MEF cells [15]. In that study, it was shown that CHOP protein controls the expression of rhodopsin during ER stress through miR-708 in the first intron of *Odz4*, a target of the UPR transcription factor CHOP. Although the study assigned a cytoprotective role to CHOP and showed that CHOP helps in the prevention of RHO overload in the ER, the role of the pro-apoptotic CHOP protein in ADRP progression has still not been discovered.

Since the T17M rhodopsin increases the pro-apoptotic CHOP protein, we investigated whether the ablation of CHOP in an ADRP retina expressing mutant opsin affects the rate of retinal degeneration. Here, we studied the role of the CHOP protein in the T17M *RHO* retina. We demonstrated that decreasing the level of CHOP does not rescue ADRP photoreceptors and, in fact, worsens the pathology. We have also discovered that the increase in the rate of retinal degeneration in ADRP mice was not linked to the over-expression of RHO as expected [15] but was instead associated with transcriptional repression in the T17M *RHO* retina.

## Materials and Methods

Five mice of each of the following genotypes were employed in this study: C57BL/6 (wild-type), CHOP−/−, T17M *RHO* CHOP+/+ (T17M *RHO*) and T17M *RHO* CHOP−/−. All groups of mice were subjected to scotopic (dark-adapted) electroretinography (ERG) and spectral domain optical coherence tomography (SD-OCT) analysis at 1, 2 and 3 months of age. In addition, retinas of 1-month old mice were collected and analyzed by quantitative RT-PCR to detect the expression of the *Nrl, Crx* and mouse *Rho* and human *RHO* genes.

### Animal Models and Protocols

Wild-type C57BL/6J mice and CHOP−/− (B6.129S-*Ddit3^tm1Dron^*/J, Stock number: 005530) were obtained from the Jackson Laboratory and were bred with T17M *RHO* mice. Homozygous CHOP−/− mice then were bred with T17M *RHO* mice to generate the T17M *RHO* CHOP−/− mice. All experimental procedures were carried out in accordance with the Institutional Animal Care and Use Committee (IACUC) protocol (Approval Number # 2009/10–45). Mice were housed in conventional cages maintained in the UNTHSC animal housing facility under specific pathogen-free conditions with cyclic light, 12-hour light: 12-hour dark at a light intensity of less than 10 lux, as measured with a light meter (model 401036; Extech, Waltham, MA). Animals were sacrificed at P30 for RNA and protein analyses.

### Scotopic Electroretinography

Mice were dark-adapted overnight, then anesthetized with ketamine (100 mg/kg) and xylazine (10 mg/kg), and their pupils were dilated in dim red light with 2.5% phenylephrine hydrochloride ophthalmic solution (Akorn, Inc.). Scotopic ERGs were recorded using a wire contacting the corneal surface with 2.5% hypromellose ophthalmic demulcant solution (Akorn.Inc). ERG was performed at different light intensities (−20 db (0.025 cd*s/m^2^), −10 db (0.25 cd*s/m^2^), 0 db (2.5 cd*s/m^2^), 5 db (7.91 cd*s/m^2^), 10 db (25 cd*s/m^2^), and 15 db (79.1 cd*s/m^2^). Five scans were performed and averaged at different light intensity. The a-wave amplitudes were measured from the baseline to the peak in the cornea-negative direction, and the b-wave amplitudes were determined from the cornea-negative peak to the major cornea-positive peak. The signal was amplified, digitized, and stored using the LKC UTAS-3000 Diagnostic System (Gaithersburg, MD).

### Spectral-Domain Optical Coherence Tomography (SD-OCT)

Mice were anesthetized with ketamine (100 mg/kg) and xylazine (10 mg/kg) and their pupils were dilated with 2.5% phenylephrine hydrochloride ophthalmic solution (Akorn.Inc). GenTeal lubricant eye gel (Novartis) and Systane Ultra Lubricant eye drops (Alcon) were used to maintain corneal hydration. Thickness of the outer nuclear layer (ONL) was measured in the superior and inferior hemispheres of the retina using the Bioptigen SD-OCT Animal Imaging Optics System. All measurements and data analysis were performed by a blinded investigator.

### Quantitative Real-Time Polymerase Chain Reaction (qRT-PCR)

For RNA extraction, whole retinas were isolated from 1-month-old mice (wild-type, T17M *RHO*, T17M *RHO* CHOP−/−, CHOP−/−) by surgical excision. Total RNA was extracted using the QIAGEN RNeasy Mini Kit. One µg of purified RNA was reverse transcribed into cDNA using iScript™ Reverse Transcription Supermix (BioRad). Integrity of the RNA samples as well as efficiency of cDNA reaction was verified prior to the qRT-PCR. TaqMan Gene Expression Assay kits (Applied Biosystems) were used to measure gene expression (Nrl: Mm00476550m1; Crx: Mm00483994m1; mRho: Mm00520345m1; hRho: Hs00892431m1; Gapdh: Mm99999915g1; Ep300:Hs00914223_m1; HDAC1: 00606262_g1; Gapdh: Hs02758991_g1). TaqMan miRNA assay was performed using the TaqMan MicroRNA Reverse Transcription kit (Applied Biosystems) and total 500 ng RNA was reverse transcribed into cDNA with specific miRNA primers (has-miR-708). Small nucleolar RNA 202 was used as a control. Quantitative real-time PCR was performed with the Step One Plus™ Real-Time PCR System (Applied Biosystems) based on the relative standard curve method. Reactions were performed at 50°C for 2 minutes and 95°C for 10 minutes, followed by 40 cycles at 95°C for 15 seconds and 60°C for 1 minute. Results were expressed as cycle threshold time (Ct) and were normalized to Ct times for the housekeeping gene *GAPDH*. The replicated RQ (Relative Quantity) values for each biological sample were averaged. Biological samples from each strain were used for qRT-PCR analyses.

### Western Blot Analysis

For protein extraction, whole retinas were isolated from 1-month-old mice (wild-type, T17M *RHO*, T17M *RHO* CHOP−/−, CHOP−/−) by surgical excision. Total protein was extracted via sonication in a protein extraction buffer containing 25 mM sucrose, 100 mM Tris-HCl, pH = 7.8, and a mixture of protease inhibitors (PMSF, TLCK, aprotinin, leupeptin, and pepstatin). Protein concentrations were determined using BioRad Protein Assays based on the Bradford method of protein quantitation. Proteins (30–40 ug) were separated in 4–20% Criterion Precast gels and 5% polyacrylamide gels (BioRad), transferred to a polyvinylidene difluoride (PVDF) membrane using the Trans-Blot Turbo Transfer System (BioRad) and incubated with primary antibodies against p-eIf2α (1∶500, Sigma-Aldrich, #SAB430022), Xbp1 (1∶200, Santa Cruz Biotechnology, #SC-32138), Hdac1 (1∶1,000, Santa Cruz Biotechnology, #SC-6299) and P300 (1∶1,000, Santa Cruz Biotechnology, #SC-584). Goat anti-rabbit (1∶10,000, #926-68021), donkey anti-goat (1∶10, 000, #926-32214), and donkey anti-mouse (1∶10,000, #926-32210) secondary antibodies were used (LI-COR Odyssey). β-actin was used as a gel loading control and was detected using an anti-β-actin antibody (1∶5000, Sigma-Aldrich, #A1978). The developed membrane was imaged using the LI-COR Odyssey Quantitative Fluorescence Imaging System.

### Histology

For Hematoxylin and Eosin (H&E) staining, 1 month-old mouse eyes were enucleated, fixed overnight at 4°C in freshly prepared 4% paraformaldehyde (PFA) in 1× phosphate buffered saline (PBS). Fixed eyes were then washed with phosphate buffered saline (PBS) to remove traces of PFA. Eyes were then sequentially immersed in 10%, 20%, and 30% sucrose solutions for at least an hour. Eye cups were then embedded in cryostat compound (Tissue TEK OCT, Sakura Finetek USA, Inc., Torrance, CA) and frozen at −80°C. 12 micron sections were obtained using the cryostat (Leica CM 1510S). Slides with sectioned right and left retinas were used for further histological analysis. Cryostat sectioned retinas were stained with H&E stain to count the number of photoreceptor nuclei. Digital images of stained retinas were captured and the central, superior and inferior regions of the retina, equally spaced from the optic nerve head were analyzed. Investigator-masked analysis of images was performed.

### Immunohistochemistry

Retinal cryosections were rinsed in PBS and blocked in 2% normal goat serum, 0.3% Triton X-100 in 0,01% BSA in PBS for 1 hour at room temperature. Anti-RHO (1D4) (University of British Columbia, Vancouver, Canada) antibody was diluted in 0.1% Triton X-100 and 1% BSA in PBS and incubated with sections overnight at 4°C. The sections were then washed three times with PBS, incubated with IgG secondary antibody tagged with Cy2 (Jackson ImmunoResearch Laboratories, West Grove, PA) diluted 1∶500 in PBS, at room temperature for 1 hour, and washed with PBS. Sections were mounted with Vectashield Mounting Medium and coverslipped.

### Statistical Analysis

Data were analyzed for statistical significance using two-way analysis of variance (ANOVA) or Student’s t-test with GraphPad Prism software 5.0. All values in each group were expressed as the mean ± SEM. All group comparisons were considered significant at *P*<0.005, *P*<0.0001, *P*<0.001, and *P*<0.01 levels.

## Results

### CHOP Protein Deficiency in the T17M *RHO* Retina Led to Diminished a-wave Amplitudes

**Figure 1:**
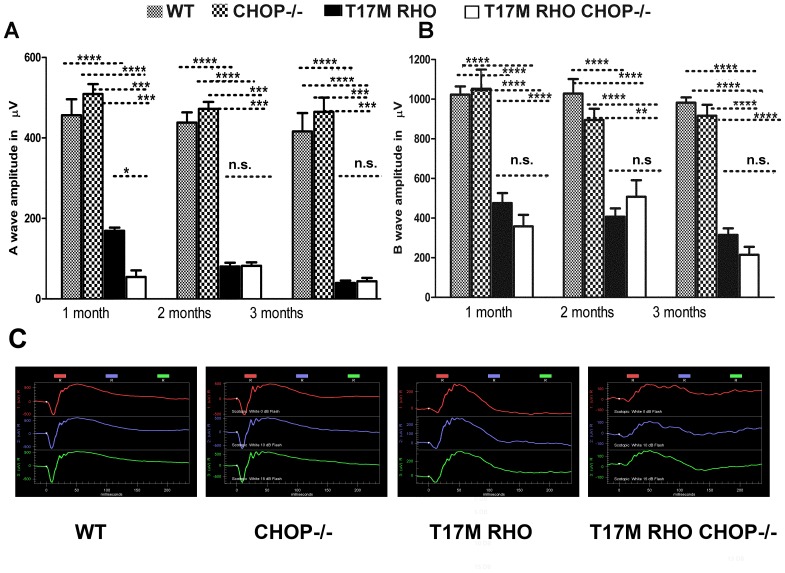
Lack of CHOP protein does not protect T17M ***RHO*** **retinas from degeneration, as measured by scotopic ERG responses at 10DB.** We analyzed 4 groups of animals (N = 6). A: The a-wave of the scotopic ERG amplitude was diminished in T17M *RHO* CHOP−/− mice at 1 month of age and the values of a-wave amplitudes were 456.3 µv ±39.7 in wild-type; 509.1 µv ±24.9 in CHOP−/−; 169.0 µv ±7.9 in T17M *RHO*; and 54.4 µv ±16.4 in T17M *RHO* CHOP−/−. This data reflected a 70% difference in the a-wave amplitudes between T17M *RHO* and T17M *RHO* CHOP−/− mice. The differences between all groups were statistically significant (*P*<0.0001). The difference between wild-type and CHOP−/− mice was not significant (n.s.). In the 2^nd^ and the 3^rd^ months, the a-wave amplitudes declined in T17M *RHO* mice but did not decline any further in T17M *RHO* CHOP−/− mice (90.7 µv ±13.5 in T17M *RHO* vs. 82.05 µv ±8.7 in T17M *RHO* CHOP−/− at 2 months and 39.7 µv ±5.7 in T17M *RHO* vs. 44.0 µv ±8.20 in T17M *RHO* CHOP−/− at 3 months). In wild-type mice, the a-wave amplitude was 438.2±25.4, vs. 472 µv ±17.3 in CHOP−/− mice at 2 months, while it was 416.2 µv ±45.5 in wild-type vs. 464.9 µv ±35.2 in CHOP−/− mice at 3 months. At 2 and 3 months, the differences between wild-type or CHOP−/− and T17M *RHO* or T17M *RHO* CHOP−/− groups were significant (*P*<0.001), but we did not register any differences between wild-type and CHOP−/− mice at 1, 2, or 3 months of age and difference between T17M RHO and T17M RHO CHOP−/− mice at 2 and 3 months of age. B: The b-wave of the scotopic ERG amplitude was decreased in T17M *RHO* CHOP−/− mice over the 3 examined months when compared to T17M *RHO* retinas. In 1-month-old animals, the b-wave amplitudes were 1023.0 µv ±41.6 in wild-type; 1052.0 µv ±96.5 in CHOP−/−; 475.6 µv ±50.7 in T17M *RHO*; and 358.2 µv ±58.2 in T17M *RHO* CHOP−/−. The differences between all groups were statistically significant (*P*<0.0001), except between T17M *RHO* and T17M *RHO* CHOP−/−, which was not significant. In the 2^nd^ and 3^rd^ months, the b-wave amplitudes in the T17M *RHO* mice declined. However, in the T17M *RHO* CHOP−/− mice, they were consistently low during the next 2 months. The b-wave amplitudes were 407.1 µv ±41.7 in T17M *RHO* vs. 428.7.5 µv ±67.0 in T17M *RHO* CHOP−/− at 2 months and 315.3 µv ±32.7 in T17M *RHO* vs. 214.9 µv ±40.0 in T17M *RHO* CHOP−/− at 3 months and were not significantly different. No difference was detected between the wild-type and CHOP−/− mice (1028.0 µv ±73.4 in wild-type vs. 894.7 µv ±56.9 in CHOP−/− at 2 months and 982.4 µv ±26.4 in wild-type vs. 916.2 µv ±55.5 in CHOP−/− at 3 months). However, at 2 months, the differences between the wild-type and T17M *RHO* or T17M *RHO* CHOP−/− mice were significant at the *P*<0.0001 level as were those between the CHOP−/− and T17M *RHO* mice, while the differences between the CHOP−/− and T17M *RHO* CHOP−/− mice were significant at the *P*<0.01 level. At 3 months, the differences between the wild-type and T17M *RHO* or T17M *RHO* CHOP−/− mice were significant at the *P*<0.00001 level, while those between CHOP−/− and T17M *RHO* or T17M *RHO* CHOP−/− were significant at the *P*<0.0001 level. The difference in the b-wave of the ERG amplitude between T17M *RHO* and T17M *RHO* CHOP−/− mice was not significant at 3 months. C: Images of the scotopic ERG amplitudes registered at 0 DB or 2.5 cd*s/m^2^ (in red), 10 DB or 25 cd*s/m^2^ (in blue) and 15 DB or 79.1 cd/m^2^ (in green) in four groups of animals.

We measured the functional effect of the ablation of CHOP protein in T17M *RHO* photoreceptors and recorded a- and b-waves of scotopic ERG amplitudes in 4 genotypes of animals: wild-type (WT), CHOP−/−, T17M *RHO* and T17M *RHO* CHOP−/−. As shown in Fig. 1A and B, 1–3 month-old T17M *RHO* mice showed a significant decrease in the a wave amplitudes compared to that of WT or CHOP−/− that were exacerbated at 1 month of age by the absence of CHOP. At this time point, the a-wave amplitudes were 456.3 µv ±39.7 in wild-type; 509.1 µv ±24.9 in CHOP−/−; 169.0 µv ±7.9 in T17M *RHO*; and 54.4 µv ±16.4 in T17M *RHO* CHOP−/− mice; the difference between all groups was significant at the *P*<0.0001 level, with the exception of the wild-type and CHOP−/− mice, which did not show a difference. In the 2^nd^ and the 3^rd^ months, the a-wave amplitude in the T17M *RHO* mice was not statistically different from that of T17M *RHO* CHOP−/− mice (90.66 µv ±13.5 in T17M *RHO* vs. 82.1 µv ±8.7 in T17M *RHO* CHOP−/− mice at 2 months and 39.66±5.7 µv in T17M *RHO* vs. 43.9±8.20 µv in T17M *RHO* CHOP−/− at 3 months). At these time points, we did not detect any difference between the wild-type and CHOP−/− mice (438.2 µv ±25.4 in wild-type vs. 472 µv ±17.3 in CHOP−/− at 2 months and 416.2 µv ±45.5 in wild-type vs. 464.9 µv ±35.2 in CHOP−/− at 3 months). At 2 and 3 months, the differences between all groups, excluding that between wild-type and CHOP−/− mice, were significant (*P*<0.001).

**Figure 2:**
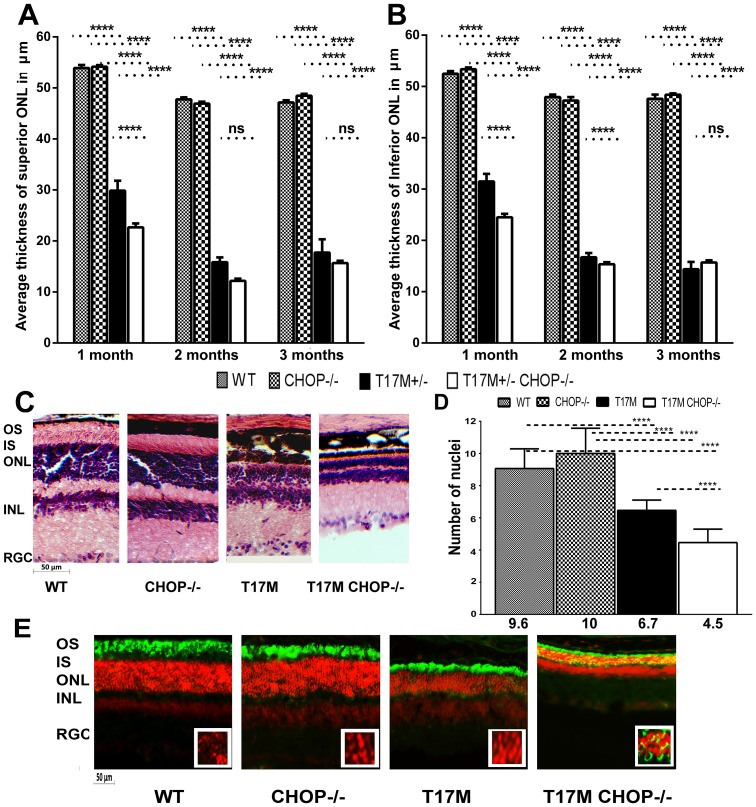
Retinal structure measured by SD-OCT was altered in the T17M ***RHO*** **CHOP−/− retina.** We analyzed 4 groups of animals (N = 6) by two-way ANOVA and found significant changes in the average thickness of the ONL in the inferior and superior hemispheres in 1,2 and 3-month-old mice. A: In the superior region, the average thickness of the ONL was 53.89 µm ±0.8 in wild-type, vs. 54.2 µm ±0.2 in CHOP−/− mice. A dramatic reduction of 22% in the ONL thickness was observed between the T17M *RHO* and T17M *RHO* CHOP−/− retinas (29.88 µm ±0.3 in T17M *RHO* vs. 23.7 µm ±0.3 in T17M *RHO* CHOP−/−), which was statistically significant (*P*<0.001). The differences between wild-type and T17M *RHO* or T17M *RHO* CHOP−/− and CHOP−/− and T17M *RHO* or T17M *RHO* CHOP−/− were also statistically significant (*P*<0.0001). No difference in the thickness of the superior ONL was observed when wild-type and CHOP−/− retinas were compared. At 2 months, the ONL thickness in the T17M *RHO* retina continued to decline and was 15.83 µm ±0.95 in T17M *RHO* vs. 12.19 µm ±0.43 in T17M *RHO* CHOP−/−), which was not statistically significant. In wild- type animals, the ONL thickness was 47.8 µm ±0.38 vs 46.92 µm ±0.37 in CHOP−/− mice. The differences between wild-type and T17M *RHO* or T17M *RHO* CHOP−/− and CHOP−/− and T17M *RHO* or T17M *RHO* CHOP−/− were also statistically significant (*P*<0.0001). No difference in the thickness of the superior ONL was observed when wild-type and CHOP−/− retinas were compared. At 3 months of age, the ONL thickness in the T17M *RHO* retina was 17.72 µm ±2.59 in T17M *RHO* vs. 15.68 µm ±0.43 in T17M *RHO* CHOP−/−), which was not statistically significant. In wild- type animals, the ONL thickness was 47.15 µm ±0.44 vs 48.47 µm ±0.37 in CHOP−/− mice. The differences between wild-type and T17M *RHO* or T17M *RHO* CHOP−/− and CHOP−/− and T17M *RHO* or T17M *RHO* CHOP−/− were also statistically significant (*P*<0.0001). No difference in the thickness of the superior ONL was observed when wild-type and CHOP−/− retinas were compared. B: The average thickness of the inferior ONL was also measured in the 4 groups of mice. We found that the average thickness was 52.5 µm ±0.51 in wild-type; 53.4 µm ±0.3 in CHOP−/− mice vs. 31.5 µm ±0.2 in T17M *RHO*; and 24.5 µm ±0.4 in T17M *RHO* CHOP−/−. The differences between wild-type and T17M *RHO* or T17M *RHO* CHOP−/− and CHOP−/− and T17M *RHO* or T17M *RHO* CHOP−/− were statistically significant (*P*<0.0001). The difference (24%) between T17M *RHO* and T17M *RHO* CHOP−/− was also statistically significant (*P*<0.001). No difference in the thickness of the inferior ONL was observed when wild-type and CHOP−/− retinas were compared. The average inferior ONL thickness in 2 month-old animals was different in all groups and was 48.0 µm ±0.46 in wild-type; 47.3 µm ±0.75 in CHOP−/− mice vs. 16.7 µm ±0.8 in T17M *RHO*; and 15.4 µm ±0.4 in T17M *RHO* CHOP−/−. The differences between wild-type and T17M *RHO* or T17M *RHO* CHOP−/− and CHOP−/− and T17M *RHO* or T17M *RHO* CHOP−/− were statistically significant (*P*<0.0001). The difference between T17M *RHO* and T17M *RHO* CHOP−/− was also statistically significant (*P*<0.001). No difference in the thickness of the inferior ONL was observed when wild-type and CHOP−/− retinas were compared. At 3 months of age the difference in the average inferior ONL thickness was not significant between T17M *RHO* and T17M *RHO* CHOP−/− mice (14.4 µm ±0.8 in T17M *RHO* vs 15.6 µm ±0.4 in T17M *RHO* CHOP−/−) while differences between wild-type (47.6 µm ±0.8) and T17M *RHO* or T17M *RHO* CHOP−/− and CHOP−/− (48.4 µm ±0.3) and T17M *RHO* or T17M *RHO* CHOP−/− were statistically significant (*P*<0.0001). No difference in the thickness of the inferior ONL was observed when wild-type and CHOP−/− retinas are compared. C: Histological analyses of wild-type, T17M RHO, T17M RHO CHOP−/− and CHOP−/− retinas: Images of wild-type, T17M RHO, T17M RHO CHOP−/− and CHOP−/− retinas stained with hematoxylin and eosin (H&E). Four animals in each group were used in this experiment. Histology of experimental mouse retinas at 1 month of age showed loss of photoreceptor cell nuclei, shortening of the outer segments, and general disorganization in the T17M RHO retina. Ablation of the CHOP protein in these retinas, however, led to more rapid retinal degeneration that resulted from the shortening of the outer and inner segments and more pronounced general retinal disorganization in the T17M RHO CHOP−/− mice. GCL, retinal ganglion cells; IPL, inner plexiform layer; INL, inner nuclear layer; ONL, outer nuclear layer; IS, inner segments; OS, outer segments. Scale bar indicates 50 µm. D: Photoreceptor cell nuclei in all 4 groups of animals. The number of nuclei was counted by a masked researcher. Two-way Anova with multiple comparison analysis demonstrated differences in all 4 groups of animals (****, P<0.0001) at 1 month of age with the exception of comparison between wild-type and CHOP−/− mice. For example, one-month-old T17M RHO mice had 6.7±0.15 rows whereas the T17M RHO CHOP−/− mice had more severe loss of photoreceptor cells with 4.5±0. 21 rows. These numbers were significantly different from those in the wild type and CHOP−/− animals (9.6±0.31 and 10.0±0.41 respectively). Histological analysis confirmed our OCT data suggesting first, that there is a decline in the number of photoreceptor cells in T17M RHO at 1month and second, the CHOP ablation expedites retinal degeneration in T17M RHO retina. Decline in the number of photoreceptors in these animals was 33% compared to T17M RHO mice. E: Immunohistological analyses of one-month-old wild-type, T17M *RHO*, T17M *RHO* CHOP−/− and CHOP−/− retinas. 12 micron cryostat sections of retinas were treated with anti-rhodopsin antibody (in green). The IHC analysis revealed normal localization of rhodopsin in the outer segments of photoreceptor cells in wild type and CHOP−/− retinas. The T17M *RHO* retinas demonstrated shortening of the OS of photoreceptors (propidium iodide -stained ONL nuclei in red). In the T17M RHO *CHOP−/−* retina we detected mislocalization of rhodopsin (in yellow) in addition to the shortening of the OS of photoreceptors. Rhodopsin was found in the cytoplasm of photoreceptors around the nuclei (in yellow ONL layer) and this, evidently indicates more severe retinal degeneration compared to T17M *RHO* mice. RGC, retinal ganglion cells; IPL, inner plexiform layer; INL, inner nuclear layer; ONL, outer nuclear layer; IS, inner segments; OS, outer segments. Scale bar indicates 50 µm.

**Figure 3:**
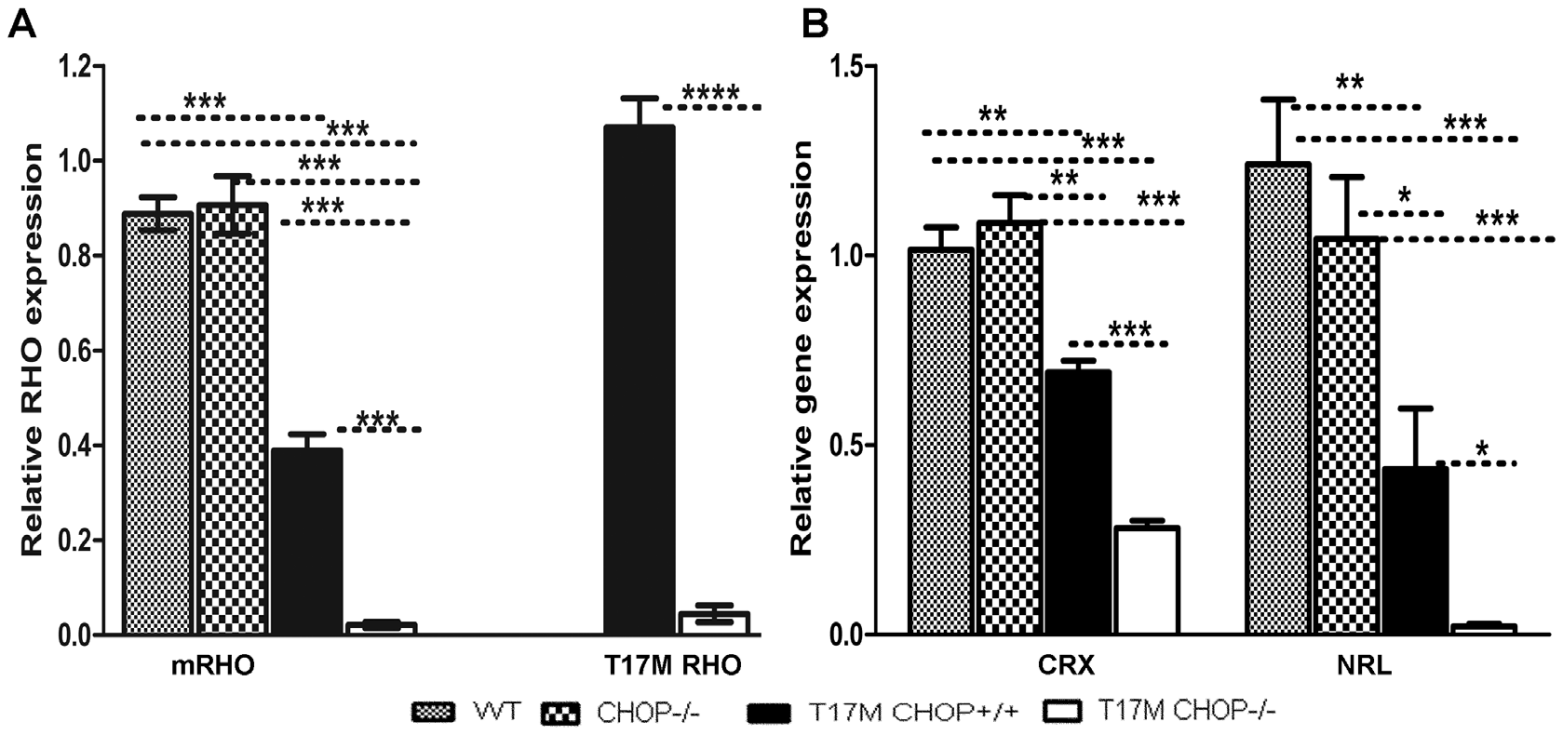
CHOP ablation in P30 T17M ***RHO*** **retinas modulates the expression of Rhodopsin (m-Rho and h-Rho) and photoreceptor-specific transcription factor (Crx and Nrl) genes.** We analyzed 4 groups of animals (N = 6) and found differences in the expression levels of *Crx*, *Nrl* and *RHO* (mRho and T17M *RHO*). A: mRho and T17M *RHO* expression was modulated in T17M *RHO* CHOP−/− retinas. The relative expression of endogenous mouse *Rho* was 0.9±0.03 in wild-type; 0.9±0.06 in CHOP−/−; 0.4±0.03 in T17M *RHO*; and 0.02±0.01 in T17M *RHO* CHOP−/− mice. The differences between wild-type and T17M *RHO* or T17M *RHO* CHOP−/− and CHOP−/− and T17M *RHO* or T17M *RHO* CHOP−/− were statistically significant (*P*<0.001). The 96% reduction of endogenous *RHO* gene expression, observed in T17M *RHO* CHOP−/− mice compared to T17M *RHO* mice was statistically significant (*P*<0.001). No difference in *Crx* gene expression was observed when the expression of mRho mRNA was compared in wild-type and CHOP retinas. The expression of the human T17M *RHO* transgene was also modulated in T17M *RHO* CHOP−/− mice, with a value of 1.1±0.06 in T17M *RHO* mice vs. 0.04±0.01 in T17M *RHO* CHOP−/− mice. The observed 96% reduction of transgene expression was statistically significant (*P*<0.0001). B: Modified Expressions of *Crx* and *Nrl* in the T17M *RHO* CHOP−/− retina. The relative *Crx* gene expression was 1.0±0.059 in wild-type; 1.1±0.1 in CHOP−/−; 0.7±0.03 in T17M *RHO;* and 0.3±0.02 in T17M *RHO*. The differences between the wild-type or CHOP−/− and T17M *RHO* groups were statistically significant (*P*<0.01), as were the differences between wild-type or CHOP−/− and T17M *RHO* CHOP−/− (*P*<0.001). The 60% reduction of *Crx* gene expression detected in T17M *RHO* CHOP−/− retinas compared to T17M *RHO* was statistically significant (P<0.001). No difference was observed when the wild-type and CHOP−/− retinas were compared. The level of *Nrl* gene expression was 1.2±0.2 in wild-type; 1.04±0.2 in CHOP−/−; 0.4±0.15 in T17M *RHO;* and 0.02±0.03 in T17M RHO CHOP−/−. The differences between wild-type and T17M *RHO* CHOP−/− and CHOP−/− and T17M *RHO* CHOP−/− were significant at the *P*<0.001 level, while those between wild-type and T17M *RHO,* T17M *RHO* and CHOP−/−, and T17M *RHO* and T17M *RHO* CHOP−/− were significant at the *P*<0.01 level. The 95% reduction of *Nrl* gene expression observed in the T17M *RHO* CHOP−/− compared to the T17M RHO retina was statistically significant (*P*<0.05). No difference in *Nrl* gene expression was observed when wild-type and CHOP−/− retinas were compared.

The b-wave amplitudes also decreased in the T17M *RHO* CHOP−/− mice over months 1–3. For example, the b-wave amplitudes in 1-month-old animals were 1023.0 µv ±41.6 in wild-type; 1052.0 µv ±96.5 in CHOP−/−; 475.6 µv ±50.7 in T17M *RHO*; and 358.2 µv ±58.9 in T17M *RHO* CHOP−/− mice. The differences between all groups, except between wild-type vs. CHOP−/− mice and T17M *RHO* vs. T17M *RHO* CHOP−/− mice, were significant at the *P*<0.0001 level. In the 2^nd^ and the 3^rd^ months, the b-wave amplitudes in T17M *RHO* and T17M *RHO* CHOP−/− mice were consistently low compared to that in wild-type or CHOP−/− mice. The b-wave amplitudes were 407.1 µv ±41.7 in T17M *RHO* vs. 428.7.5 µv ±67.0 in T17M *RHO* CHOP−/− mice at 2 months and 315.3±32.7 µv in T17M *RHO* vs. 214.9 µv ±40.00 in T17M *RHO* CHOP−/− mice at 3 months, though these differences were not significant. We also did not detect any difference between wild-type and CHOP−/− mice (1028.0 µv ±73.4 in wild-type vs. 894.7 µv ±56.9 in CHOP−/− mice at 2 months and at 3 months (982.4±26.5 µv in wild-type vs. 916.2 µv ±55.5 in CHOP−/−). At 2 months, the difference between wild-type and T17M *RHO* or T17M *RHO* CHOP−/− was significant at the *P*<0.0001 level, as was the difference between CHOP−/− and T17M *RHO*, while the difference between CHOP−/− and T17M *RHO* CHOP−/− was significant at the *P*<0.01 level. At 3 months, the difference between wild-type and T17M *RHO* or T17M *RHO* CHOP−/− mice was significant at the *P*<0.0001 level, as was the difference between CHOP−/− and T17M *RHO* or T17M *RHO* CHOP−/−. The differences in the b-waves of the ERG amplitudes between the T17M *RHO* and T17M *RHO* CHOP−/− mice were not significant during these 3 months (n.s.).

We also analyzed the a- and b-wave implicit times for T17M *RHO* CHOP−/− mice by two-way Anova (Fig. S1) and found that at 1 month of age the a-wave implicit time was increased by 70% in T17M *RHO* CHOP−/− mice compared to all other groups (8.7±0.4 ms in T17M *RHO* vs 14.9±4.4 ms in T17M *RHO* CHOP−/− vs 8.6±0.4 ms in wild-type vs 8.4±0.3 ms in CHOP−/− mice). However, by 3 months of age the difference between the T17M *RHO* CHOP−/− and all others was not that dramatic and was significant (*P*<0.01) only when compared to CHOP−/− retinas (9.5±0.8 ms in T17M *RHO* vs 12.13±0.9 ms in T17M *RHO* CHOP−/− vs 8.3±0.2 ms in wild-type vs 7.3±0.2 ms in CHOP−/− mice). Interesting, no difference was observed between T17M *RHO* and wild-type at 1, 2 and 3 months of age when compared the T17M *RHO* to all other groups of animals. However, the exclusion of T17M *RHO* CHOP−/− mice from the comparison demonstrated statistical difference between T17M *RHO* mice and wild-type or CHOP−/− mice at 2 and 3 months (** *P*<0.01).

Analysis of the b-wave implicit time demonstrated no difference between all groups at 1, 2 and 3 months of age for the exclusion of 3 month-old T17M *RHO* CHOP−/− retina that had a 40% increase in the implicit time for the b-wave amplitude compared to T17M RHO retina. At 3 months the b-wave implicit time was 50.31±2.12 ms in T17M RHO vs 36.17±5.24 ms in T17M RHO CHOP−/− vs 26.67±1.76 ms in wild-type vs 31.81±0.76 ms in CHOP−/− mice.

### CHOP Protein Deficiency in the T17M *RHO* Retina Led to a Reduction in the Thickness of the Outer Nuclear Layer

We also conducted measurements of the average thickness of the Outer Nuclear Layer (ONL) (400 µm from the Optic Nerve Head in superior and inferior regions) in all 4 groups of mice (Fig. 2 and Fig. S2) and found that the average thicknesses of the ONL in both hemispheres in wild-type and CHOP−/− mice were not different at 1, 2 and 3 months. At 1 month of age in the superior region, we recorded a thickness of 53.9 µm ±0.8 in wild-type vs. 54.2 µm ±0.2 in CHOP−/− mice. In the inferior region, we found that the thickness was 52.5 µm ±0.2 in wild-type vs. 53.5 µm ±0.3 in CHOP−/− mice. As expected [4], retinal degeneration in the T17M *RHO* retinas led to a dramatic reduction in the average ONL thickness (29.9 µm ±0.3 in the superior region and 31.5 µm ±0.2 in the inferior region). However, the T17M *RHO* retinas deficient in CHOP protein showed a more dramatic reduction (by 24–22%) in the ONL thickness in both the superior (23.7 µm ±0.3) and inferior hemispheres (24.5 µm ±0.4) compared to the T17M *RHO* retinas. At 1 month the difference between all four groups were statistically different at the *P*<0.001 level. Over the next month the average inferior ONL thickness was significantly different in T17M *RHO* CHOP−/− compared to T17M *RHO* retinas and no difference was detected in the superior ONL thickness. At 3 months, despite the fact that the T17M *RHO* retina continued to degenerate neither the inferior or superior ONL in T17M *RHO* retinas were not statistically different in T17M *RHO* CHOP−/− compared to T17M *RHO*. At 2 and 3 months the difference between wild-type vs. T17M *RHO* or T17M *RHO* CHOP−/− mice and CHOP−/− vs. T17M *RHO* or T17M *RHO* CHOP−/− mice were different at the *P*<0.001 level.

Retinal histological analysis of four groups of mice demonstrated severe retinal degeneration in T17M *RHO* mice compared to wild-type or CHOP−/− mice. The T17M *RHO* CHOP−/− retinas were characterized by more pronounced retinal degeneration even when compared to T17M *RHO* mice. The inner and outer segments of T17M *RHO* CHOP−/− photoreceptors looked significantly shorter and the length of the outer nuclear layer seems to be dramatically reduced (Fig. 2C). The number of the nuclei was also different (Fig. 2D). While wild-type and CHOP−/− mice did not differ in the numbers of photoreceptors in presented areas, the T17M *RHO* mice showed a 31% reduction in the number of photoreceptors compared to wild-type and a 49% increase compared to T17M *RHO* CHOP−/−.

Immunohistological analysis of the retinas (Fig. 2E) revealed that the transmembrane rhodopsin protein had aberrant trafficking in the T17M *RHO* CHOP−/− photoreceptors and accumulated in the cytoplasm of photoreceptors near the nucleus. It also confirmed a similarity in the integrity of retinal structure in the wild-type and CHOP−/− mice.

### CHOP Protein Deficiency in the T17M *RHO* Retina Led to a Decrease in Mouse and Human *RHO* mRNA

From our previous study, we knew that the T17M *RHO* retinas experience activation of ER stress and increase in the CHOP mRNA at earlier stage of ADRP and decrease in RHO protein content [4]. However, not significant difference in the CHOP expression has been found in adult T17M *RHO* mice compared to wild-type at P25. We also learned that the *CHOP* gene controls the expression of *RHO* mRNA during ER stress through transcriptional regulation of miR-708, which is known to be increased under induced UPR [15]. Therefore, we hypothesized that the absence of the CHOP protein in the T17M *RHO* retina and consequently, the lack of a mechanism regulating RHO expression during ER stress would lead to over-production of rhodopsin protein in photoreceptors, triggering their death [16]. Thus, anticipating that over-expression of *RHO* mRNA and protein would be observed, we performed qRT-PCR (Fig. 3) and western blot analyses. Unexpectedly, we observed decreases in both mouse and human *RHO* mRNA in T17M *RHO* CHOP−/− retinas.

We previously reported that the level of *mRHO* mRNA expression is diminished in T17M *RHO* compared to wild-type [4]. In the present study, we observed a 57% reduction of endogenous mouse *Rho* mRNA expression in T17M *RHO* compared to wild-type retinas and a 96% reduction of *RHO* mRNA in T17M *RHO* CHOP−/− retinas compared to T17M *RHO* retinas (0.9±0.03 in wild-type; 0.9±0.06 in CHOP−/−; 0.4±0.3 in T17M *RHO*; and 0.02±0.006 in T17M *RHO* CHOP−/−. The *P* value was <0.0001 in all groups except for the wild-type and CHOP−/− mice. In these groups, the difference was not significant. Surprisingly, in T17M *RHO* CHOP−/− retinas, we observed an even more dramatic reduction in the expression of both the endogenous mouse *RHO* mRNA and the human *RHO* transgene. The human *RHO* transgene was reduced by 96% in T17M *RHO* CHOP−/− mice compared to T17M *RHO* mice (1.07±0.06 in T17M RHO vs. 0.04±0.02, *P* = 0.0001).

### CHOP Protein Deficiency in the T17M *RHO* Retina Led to a Decrease in the Photoreceptor-Specific Transcriptional Factors, Nrl and Crx

In an attempt to determine what might cause the observed down-regulation of mouse and human *RHO* mRNA expression, we analyzed the transcriptional factors, Nrl and Crx (Fig. 3), which have previously been shown to be down-regulated in ADRP transgenic retinas [4], [14]. No significant difference in the patterns of expression of the two transcriptional factors was detected between wild-type and CHOP−/− mice. However, the difference in *Crx* and *Nrl* gene expression, normalized to the housekeeping gene *GAPDH*, between the T17M *RHO* and T17M *RHO* CHOP−/− mice was dramatic. For example, for the *Crx* gene, we recorded values of 1.0±0.06 in wild-type; 1.1±0.1 in CHOP−/−; 0.7±0.03 in T17M *RHO*; and 0.3±0.02 in T17M *RHO* CHOP−/−. The differences between groups were significant at the *P*<0.001 level. One exception was the difference between wild-type and T17M *RHO* (*P*<0.01). Therefore, qRT-PCR analysis of *Crx* gene expression demonstrated a 60% reduction of its mRNA level in the T17M *RHO* CHOP−/− retina compared to the T17M *RHO* retina.

The relative *Nrl* gene expression level was 1.2±0.2 in wild-type; 1.04±0.2 in CHOP−/−; 0.4±0.2 In T17M *RHO;* and 0.02±0.03 in T17M RHO CHOP−/−. The differences between the wild-type and T17M *RHO* CHOP−/− and between the CHOP−/− and T17M *RHO* CHOP−/− groups were significant at the *P*<0.001 level, whereas those between the wild-type and T17M *RHO,* T17M *RHO* and CHOP−/−, and T17M *RHO* and T17M *RHO* CHOP−/− mice were significant at the *P*<0.01 level. The differences between the wild-type and CHOP−/− mice were not statistically significant. Therefore, qRT-PCR analysis of *Nrl* gene expression demonstrated a 95% reduction in the T17M *RHO* CHOP−/− retina compared to the T17M RHO retina.

Next, we attempted to understand the cause of modulation of the RHO mRNA and performed the experiment in which we analyzed the level of miR-708 (Fig. S3). Comparing RQs from all four groups of one-month-old retinas by one-way Anova we found no difference between animals. We also did not detect any difference between wild-type and CHOP−/− or wild-type and T17M *RHO* mice by t-test comparison. However, when the CHOP−/− and T17M *RHO* CHOP−/− mice were compared by t-test analysis the difference was statistically significant (*P*<0.05).

### CHOP Protein Deficiency in the T17M *RHO* Retina Led to an Increase in p-eIf2**α** and Decrease in sXbp1 Protein

**Figure 4:**
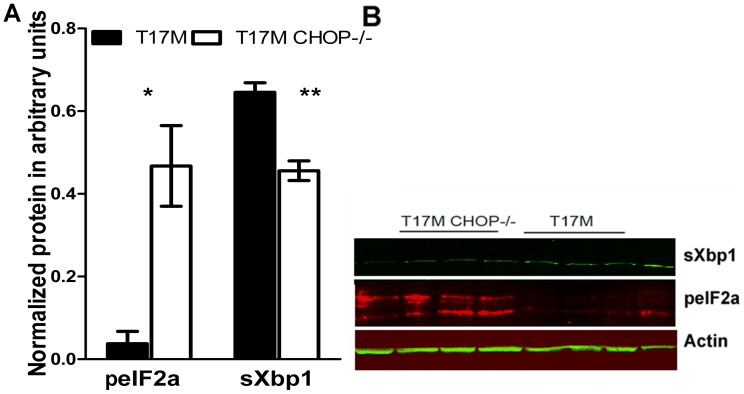
Ablation of CHOP protein in P30 T17M ***RHO*** **retinas led to modulation of the PERK and IRE1 pathways of the UPR.** A: We analyzed T17M *RHO* and T17M *RHO* CHOP−/− retinas (N = 4) and found that the expression of phosphorylated eIF2α protein was increased by over 8 fold in T17M *RHO* CHOP−/− mice (0.5±0.1 a.u.vs. 0.04±0.1 a.u., *P* = 0.01). The level of spliced Xbp1 in the T17M *RHO* retina was decreased by 30%, presenting a value of 0.64±0.027 a.u. vs. 0.4±0.03 a.u.in T17M *RHO* CHOP−/− mice (*P* = 0.004). B: Representative images of western blots treated with antibodies against Xbp1, peIF2α and β-actin proteins.

We did not detect any differences in retinal structure and function between wild-type and CHOP−/− retinas. Therefore, we further proceeded to compare the T17M *RHO* and T17M *RHO* CHOP−/− retinas and search for the cellular signaling mechanism responsible for the severe retinal degeneration observed in T17M *RHO* CHOP−/− mice. From our previous study [4], we knew that the activation of the UPR in the P15 T17M *RHO* retina leads to phosphorylation of the eIF2a protein. In addition, from the literature, we learned that in the CHOP−/− hearts with transverse aortic constriction mimicking human heart failure leading to activated ER stress and ER stress-induced apoptosis, an increased peIf2a level is observed [12]. Therefore, we examined p-eIf2α in T17M *RHO* CHOP−/− retinas (Fig. 4A and B) and found that the level of peIf2α was over 8-fold higher in T17M *RHO* CHOP−/− mice compared to T17M *RHO* mice (0.5±0.1 arbitrary units (a.u.) vs. 0.04±0.1 a.u., *P* = 0.01).

Another UPR pathway found to be up-regulated during the UPR in T17M *RHO* mice [4] is Xbp1 signaling, which is known to generally serve as a pro-survival arm of the UPR [17]. The spliced form of Xbp1 (sXbp1) is a transcription factor that up-regulates a series of UPR related chaperone proteins [18]. Therefore, we wondered what might happen to the splicing of Xbp1 in the absence of C\EBP homologous protein. The results of protein analysis demonstrated that the level of spliced Xbp1 in the T17M *RHO* retina was reduced by 30% to 0.6±0.03 a.u., whereas the level in T17M *RHO* CHOP−/− mice was 0.4±0.03 a.u, (*P* = 0.004).

### CHOP Protein Deficiency in the T17M *RHO* Retina Led to Enhanced Expression of Hdac1 Protein and Reduced P300 Protein Expression

**Figure 5:**
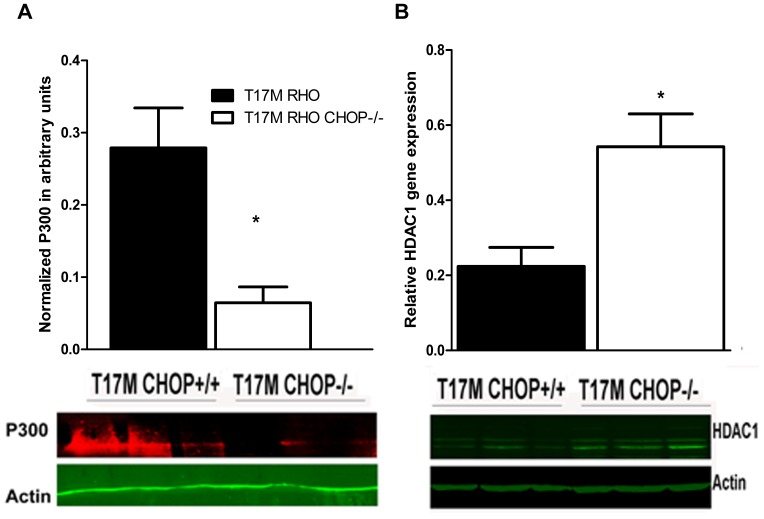
Expressions of the histone deacetylase, Hdac1 and the transcriptional co-activator, P300 were modified in P30 T17M ***RHO*** **CHOP−/− retinas (N = 3).** A: A 78% reduction in the P300 protein level was observed in the T17M *RHO* CHOP−/− retina compared to T17M *RHO*. The normalized level of P300 was 0.3±0.06 a.u. in T17M *RHO* vs. 0.1±0.02 a.u. in T17M *RHO* CHOP−/− (P = 0.02). B: A 245% increase in Hdac1 protein expression was observed in T17M *RHO* CHOP−/− retinas (0.22±0.05 arbitrary units in T17M *RHO* vs. 0.54±0.08 a. u. in T17M *RHO* CHOP−/−, *P* = 0.03). Bottom: Representative images of western blots treated with antibodies against P300, HDAC1 and β-actin proteins.

When we were searching for the link leading to down-regulation of *Nrl*, *Crx* and consequent down-regulation of *RHO* gene expression in the T17M *RHO* CHOP−/− mice, we found that critical regulation of activity, or even degradation of the ER stress–induced CHOP transcriptional factor occurs through binding of P300/Cbp-associated factor at the N-terminal domain [19]. The same P300/Cbp complex binds to the Crx transcriptional factor to initiate the expression of photoreceptor-specific genes, including rhodopsin [20]. Therefore, we next analyzed the expression of the histone acetyl**-**transferase, P300 and histone deacetylase, Hdac1, which is known to be a binding partner for the CHOP transcriptional factor [19], in T17M *RH*O CHOP−/− protein extracts (Fig. 5A and B).

In this experiment, we observed a 78% reduction in the P300 protein level in the T17M *RHO* CHOP−/− compared to that in the T17M *RHO* retina. For example, the normalized level of the P300 protein was 0.3±0.1 a.u.in T17M *RHO* arbitrary units vs. 0.1±0.02 a.u.in T17M *RHO* CHOP−/− arbitrary units (*P* = 0.02). In contrast, Hdac1 protein, was significantly increased by 245% in T17M *RHO* CHOP−/− retinas compared to retinas from T17M *RHO* mice. The level was 0.2±0.05 a.u. in T17M *RHO* and 0.5±0.1 a.u., in T17M RHO CHOP−/− (*P* = 0.03).

## Discussion

CHOP protein is known to be over-expressed in ADRP photoreceptors [4], [13], [14] and is a major factor, the ablation of which leads to attenuation of pathogenic hallmarks of different degenerative disorders [11], [21], [22]. Therefore, it was logical for us to test the hypothesis that creating a deficiency in CHOP protein could be used as a therapy for ADRP photoreceptors. Thus, in this study, we not only demonstrated that CHOP protein could not be considered as a potential therapeutic target for treating ADRP photoreceptors but also revealed the cause of accelerated retinal degeneration in ADRP retinas deficient in CHOP.

Despite the fact that the T17M *RHO* retina is known to rapidly degenerate [4], the T17M *RHO* CHOP−/− retinas demonstrated a much more severe loss of the a-wave amplitudes of the scotopic electroretinogram. For example, in 1-month-old T17M *RHO* CHOP−/− retinas, we observed a 76% loss of a-wave amplitude compared to T17M *RHO* mice and no significant difference in the b-wave amplitude, suggesting first, that the main physiological changes occur in the T17M *RHO* CHOP−/− photoreceptors and second, that the ADRP photoreceptor cells experiencing the activation of the UPR due to expression of the human T17M *RHO* transgene respond to CHOP ablation more rapidly than bipolar cells. Despite the fact that the reduction of ERG b-wave amplitudes shows just one trend in the T17M *RHO* CHOP−/− retina, the loss of photoreceptors is evidently responsible for the lessening of b-wave amplitude and the fact that the CHOP−/− retinas do not demonstrate loss of either a- or b-wave amplitudes and do exhibit normal ERG favors this hypothesis. In 2- and 3-month-old T17M *RHO* and T17M *RHO* CHOP−/− mice, both the a- and b-wave amplitudes were almost identical because the T17M *RHO* retinas fully degenerate by this time point. In 1 month-old mice, the measurement of implicit time of the a- wave ERG amplitudes also reveals that it is was significantly longer in T17M *RHO* CHOP−/− mice compared to all other groups and indicates a delay in the response of photoreceptors to the light flash (*P*<0.001). The implicit time for the b-wave ERG amplitude is changed slowly in T17M *RHO* CHOP−/− mice and only by 3 month of age. The response of bipolar cells to the light stimulation of photoreceptors is postponed. This fact indicates that despite no differences in the b-wave ERG amplitudes between T17M *RHO* and T17M *RHO* CHOP−/− mice is observed, the bipolar cells respond slower suggesting that functional changes in these mice could be obvious at later time points.

The functional loss of photoreceptors in T17M *RHO* CHOP−/− retinas, which is already accelerated by 1 month, could be a result of photoreceptor cell death, which we confirmed by SD-OCT, histological and IHC analyses. In the T17M *RHO* CHOP−/− retinas, the average thickness of the ONL in the superior and inferior regions were reduced by 24% and 22%, respectively. The number of nuclei in the ONL was dramatically reduced by 33% suggesting that in these animals, the photoreceptor cells were undergoing morphological changes. In addition, the ablation of CHOP protein in T17M *RHO* retina at least partially prevents the RHO protein from its trafficking to the OS and promotes its aggregation increasing the burden of misfolded protein in photoreceptors. At this point we do not know a precise localization of mistrafficking rhodopsin. To answer the question of whether rhodopsin retains within the Endoplasmic Reticulum or forms agglomerates in the cytosol of PR, additional experiments such as electron microscopy should be conducted.

Therefore, the photoreceptor cell death could be hastened in T17M *RHO* CHOP−/− mice by modified cellular signaling, thus leading to advanced retinal degeneration by 1 month of age. Therefore, we next performed RNA and protein analyses.

Behram et al. [15] noted the relationship between the *RHO* and CHOP genes at the level of post-transcriptional regulation of *RHO* mRNA during activation of the UPR. That study revealed that this control occurs through transcriptional regulation of miR-708 by CHOP protein and assigned the CHOP protein a cytoprotective function. Despite the significant contribution of this study to progressing our understanding of mammalian gene expression networks, the precise role of CHOP protein ablation in ADRP retinas experiencing activation of the UPR has not been studied. Therefore, we performed experiment in which we analyzed the level of miR-708 in ADRP retina. We found no difference between all four groups of animals when analyzed by one-way Anova suggesting that in ADRP retina: 1) the CHOP ablation does not promote modulation of the miR-708 expression (T17M *RHO* CHOP+/+ vs T17M *RHO* CHOP−/−) as it has been proposed for the MEFs treated with thapsigardin [15] and 2) the ER stress (T17M *RHO*) down-regulates the level of miR-708 in the CHOP deficient retina (CHOP−/− vs T17M *RHO* CHOP−/−). Overall, the conclusion we made supports the general finding presented by Behram et al. [15] that the combination of ER stress and CHOP ablation promotes reduction of the miR-708 (wild-type vs T17M RHO CHOP−/−, P = 0.03).

While looking for the cellular mechanism involved in the rapid retinal degeneration observed in T17M *RHO* CHOP−/− mice, we tested the RHO mRNA expression and found that the levels of mouse and human *RHO* mRNA were significantly reduced in T17M *RHO* CHOP−/− compared to T17M *RHO* retinas, suggesting that either photoreceptor cell death or transcriptional inhibition is responsible for this down-regulation. It also points the fact that the additional regulatory pathways besides the transcriptional regulation of the RHO by miR-708 could be responsible for accelerated retinal degeneration of T17M *RHO* CHOP−/− mice.

From a previous study by our group [4], we knew that compared to wild-type mice, T17M *RHO* mice express the photoreceptor transcriptional factors Nrl and Crx at lower levels and exhibit a major collapse of photoreceptors at P25. Therefore, we then studied *Nrl* and *Crx* gene expression.

It is known that while Crx activity regulates the differentiation of both rods and cones, the Nrl transcriptional factor is preferentially expressed in rod photoreceptors, where it is thought to act synergistically with Crx to regulate rhodopsin expression. Mutations in human *Nrl* have been associated with ADRP, which is characterized by rod photoreceptor degeneration, and deletion of *Nrl* in mice results in complete loss of rod function [23], [24]. In our experiment, we found that down-regulation of these transcriptional factors in T17M *RHO* CHOP−/− mice is probably responsible for the reduction of *RHO* expression in these mice. The observed down-regulation of *Nrl* and *Crx* by 95% and 60%, respectively, indicates that the ablation of the CHOP protein accelerates the degeneration of rod photoreceptors. It also points out the fact that other relevant phototransduction cascade genes could be down-regulated in T17M *RHO* CHOP−/− leading to significant vision loss.

The results of the ERG and OCT experiments, as well as the results of histological analysis and analysis of *RHO*, *Nrl* and *Crx* expression, show that under normal conditions, the CHOP−/− retina is not significantly different from that of wild-type mice. Therefore, we further investigated only two groups of animals: T17M *RHO* and T17M *RHO* CHOP−/− mice. Since PERK and the IRE1 signaling pathways are activated in P15 T17M *RHO* retinas [4], we examined these pathways in 1-month-old T17M *RHO* CHOP−/− retinas. We detected significant up-regulation of peIF2α in the P30 T17M *RHO* CHOP−/− retina, which suggests that these animals experience long-lasting transcriptional inhibition of gene expression in their photoreceptors. The fact that CHOP ablation leads to the over-production of peIF2α has been demonstrated previously [12]. For example, in cardiomyocytes experiencing activation of the UPR, the ablation of CHOP protein leads to an increased peIF2α level, which is considered to be cytoprotective. Under normal conditions, CHOP protein controls the expression of GADD34 that binds the protein phosphatase 1 and, thus, negatively regulates the phosphorylation of eIF2α, leading to reduced protein translation and the inhibition of protein synthesis. Therefore, under CHOP-deficient conditions, decreased expression of GADD34 enhances the phosphorylation of eIF2 α, and this lack of GADD34 is likely to contribute to the prevention of the suppression of protein synthesis. We believe that the inhibition of protein synthesis is long lasting in the T17M *RHO* CHOP−/− retina. In T17M *RHO* retinas, peIF2α is found to be up-regulated by P15. In P30 T17M *RHO* CHOP−/− retinas, this up-regulation most likely leads to transcriptional protein repression and retinal degeneration. Transcriptional inhibition of gene expression from P15 until P30, which is assumed to be the period required to successfully resolve the compromised ER homeostasis, is extended and presumably affects general biosynthesis.

Spliced XBP1 regulates genes that are implicated in protein folding, trafficking, and secretion and thus contributes to the restoration of ER homeostasis and favors cell survival under ER stress [17]. In earlier work, we demonstrated splicing of Xbp1 and consequent activation of the IRE1 pathway in T17M *RHO* retinas [4]. In the current study, we found that in T17M *RHO* CHOP−/− retinas, the level of spliced Xbp1 was decreased compared to controls, suggesting that the function of the pro-survival arm is compromised in these animals. Therefore, protein analysis of T17M *RHO* CHOP−/− retinas demonstrates an elevated level of pro-apoptotic PERK signaling and diminished level of the pro-survival Xbp1 UPR arm. Our results were consistent with the findings in a previous study in which ER stress was induced in hippocampus of the Wild-type and CHOP−/− mice by intracerebroventricular injection with tunicamycin. According to that study, CHOP−/− mice showed enhanced hippocampal cell apoptosis and decreased sXBP-1 expression [25].

To examine the link between the down-regulation of *Nrl* and *Crx* gene expression and CHOP ablation in the T17M *RHO* CHOP−/− retina, we examined the histone acetyltransferase P300, which is known to be recruited by the CHOP transcription factor [19] and to regulate opsin expression along with Crx, Nrl and PolII [20]. Additionally, dynamic histone acetylation is controlled by HDACs, which exert an opposite function and are often associated with reduced accessibility of the transcriptional machinery to genes [26].

The 1-month-old T17M *RHO* CHOP−/− retina exhibited a 2-fold higher Hdac1 protein content, suggesting that deacetylation processes prevailed in these mice. Increased histone deacetylation could repress general transcription and accelerate retinal degeneration in these mice. For example, it is known that the hypoacetylation is a major factor associated with severe retinal degeneration in rd1 mice [27]. The peak of Hdac activity occurs in rd1 mice at P11, while the peak of TUNEL staining takes place at P13 in the rd1 retina, implying that the observed photoreceptor cell death results from elevated Hdac activity. In addition, it has been shown that HdacI/II inhibitors protect rd1 photoreceptors and strongly reduce photoreceptor cell death by decreasing poly-ADP-ribose-polymerase (PARP).

In addition to an increase in Hdac1 protein expression, we found that the protein level of P300 was decreased in T17M *RHO* CHOP−/− retinas, implying that there was negative regulation between CHOP and P300 gene expression. Additionally, the reduction of spliced Xbp1 expression could also contribute to the reduction of P300 expression, as a direct dose-dependent link has been proposed between Xbp1 and P300 [28].

Despite the multiple studies focusing on the silencing of pro-apoptotic CHOP protein for the purpose of developing cellular therapies, we demonstrated that CHOP protein is a survival factor for rod photoreceptors carrying a severe ADRP mutation. The ablation of CHOP protein most likely extends transcriptional inhibition and leads to a reduction of the expression of photoreceptor-specific transcriptional factors and RHO protein, along with an increase in histone deacetylation. These findings suggest that CHOP plays a protective role in rod photoreceptor cells of ADRP affected mouse retinas. However, the role of CHOP protein in other cell types of ADRP mouse retinas needs to be clarified in the future.

## Supporting Information

Figure S1 **Mean (±SD) implicit time of the a-wave and b-wave recorded from all four groups of mice.** Dark-adapted responses (flash luminance at 10 DB) were recorded from six animals. A: Analysis of the a-wave implicit time (IT) in 1-month-old animals demonstrated significant difference between mice. For example, in the wild-type, the a-wave IT was 8.62±0.43 vs 8.63±0.36 in CHOP−/−; 8.7±0.41 in T17M *RHO* and 14.86±3.29 in T17M *RHO* CHOP−/−. The difference in a-wave IT between T17M *RHO* CHOP−/− and wild-type or T17M *RHO* CHOP−/− and T17M *RHO* or T17M *RHO* CHOP−/− and CHOP−/− strains was significant (*** *P value* <0.001). There was no difference in the a-wave IT between wild-type and CHOP−/− or wild-type and T17M RHO or CHOP−/− and T17M RHO strains. However, next two months the a-wave IT in the CHOP−/− mice dropped and was only significant compared to T17M *RHO* animals at 3 months (*** *P value* <0.001). The a-wave IT at 3 months was 8.33±0.21 in wild-type, 7.31±0.16 in CHOP−/−, 9.5±0.85 in T17M *RHO* and 12.13±0.97 in T17M *RHO* CHOP animals. B: Analysis of b-wave IT demonstrated no significant difference in all four groups of mice at 1 and 2 months of age. However, at 3 months of age the difference in the b-wave IT was significantly increased in the T17M RHO CHOP−/− mice compared to all remaining strains and was 25.67±1.76 in the wild-type; 31.81±0.76 in CHOP−/−; 37.17±5.39 in T17M *RHO* and 50.31±2.12 in T17M *RHO* CHOP−/− mice (* *P* value <0.01, *** *P* value <0.001).
(TIF)

Figure S2 **Alteration of retinal structure in the T17M** ***RHO*** **CHOP−/− retinas.** A: SD-OCT images were captured in 1-month-old live mice. B: Thickness of the Outer Nuclear Layer (ONL) was measured in the superior and inferior retinal hemispheres in 1-month old mice. Measurements were made at a distance of 100, 200, 300 and 400 µm from the optic nerve head. The ONL in the T17M *RHO* and T17M *RHO* CHOP−/− retinas was significantly thinner compared to that of wild-type or CHOP−/− retinas at corresponding time points. Also, significant difference (*** *P* value <0.001) in ONL thickness was observed between the T17M *RHO* and T17M *RHO* CHOP−/− retinas. RNF&GCL: retinal nerve fiber and ganglion cell layer; INL: Inner nuclear layer, OPL: Outer plexiform layer, ONL: Outer nuclear layer, OLM: Outer limiting membrane, IS/OS; inner and outer segments of photoreceptors and RPE: retinal pigment epithelium.
(TIF)

Figure S3 **Ablation of the CHOP protein in ADRP retina does not modulate the miR-708 expression.** We analyzed all four groups of one-month-old mice (N = 3) and found no difference between them when compared by one-way Anova. However, *T*-test comparison revealed that there is a statistically significant difference between CHOP−/− and T17M RHO CHOP−/− mice suggesting that the ER stress promotes reduction of miR-708 in CHOP deficient retinas.
(TIF)
