# Supplementary figures and images for: Ablation of C/EBP Homologous Protein Does Not Protect T17M RHO Mice from Retinal Degeneration
Source: PLoS One. 2013 Apr 30;8(4):e63205. doi: 10.1371/journal.pone.0063205 (PMC3640035; doi:10.1371/journal.pone.0063205)

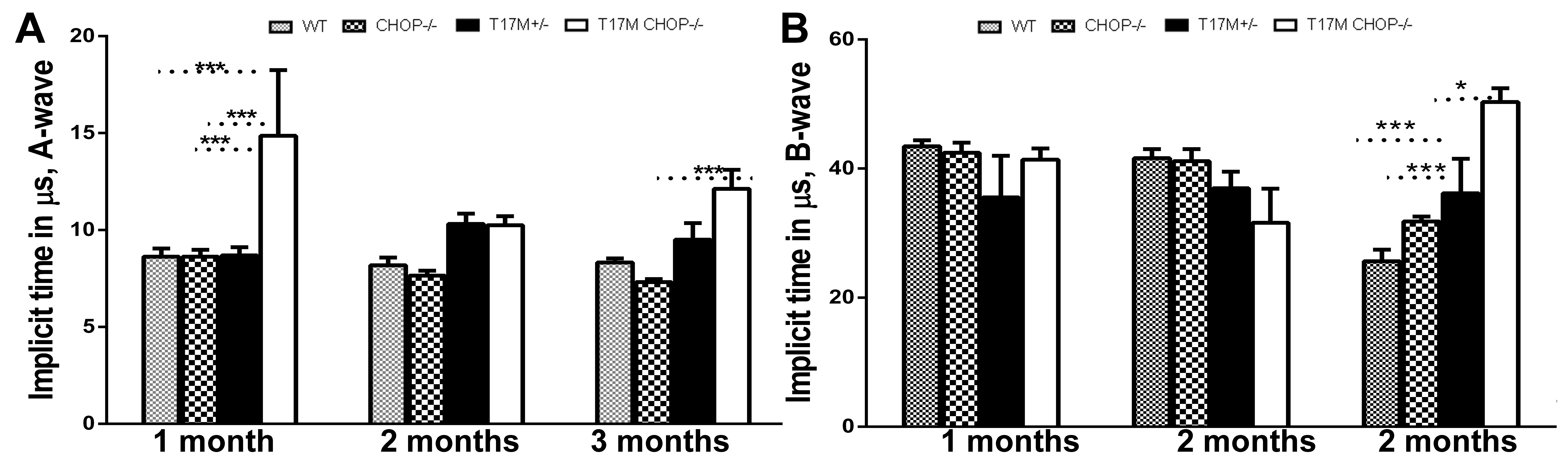

Supplement: Figure S1 — Mean (±SD) implicit time of the a-wave and b-wave recorded from all four groups of mice. Dark-adapted responses (flash luminance at 10 DB) were recorded from six animals. A: Analysis of the a-wave implicit time (IT) in 1-month-old animals demonstrated significant difference between mice. For example, in the wild-type, the a-wave IT was 8.62±0.43 vs 8.63±0.36 in CHOP−/−; 8.7±0.41 in T17M RHO and 14.86±3.29 in T17M RHO CHOP−/−. The difference in a-wave IT between T17M RHO CHOP−/− and wild-type or T17M RHO CHOP−/− and T17M RHO or T17M RHO CHOP−/− and CHOP−/− strains was significant (*** P value <0.001). There was no difference in the a-wave IT between wild-type and CHOP−/− or wild-type and T17M RHO or CHOP−/− and T17M RHO strains. However, next two months the a-wave IT in the CHOP−/− mice dropped and was only significant compared to T17M RHO animals at 3 months (*** P value <0.001). The a-wave IT at 3 months was 8.33±0.21 in wild-type, 7.31±0.16 in CHOP−/−, 9.5±0.85 in T17M RHO and 12.13±0.97 in T17M RHO CHOP animals. B: Analysis of b-wave IT demonstrated no significant difference in all four groups of mice at 1 and 2 months of age. However, at 3 months of age the difference in the b-wave IT was significantly increased in the T17M RHO CHOP−/− mice compared to all remaining strains and was 25.67±1.76 in the wild-type; 31.81±0.76 in CHOP−/−; 37.17±5.39 in T17M RHO and 50.31±2.12 in T17M RHO CHOP−/− mice (* P value <0.01, *** P value <0.001). (TIF) [file pone.0063205.s001.tif]

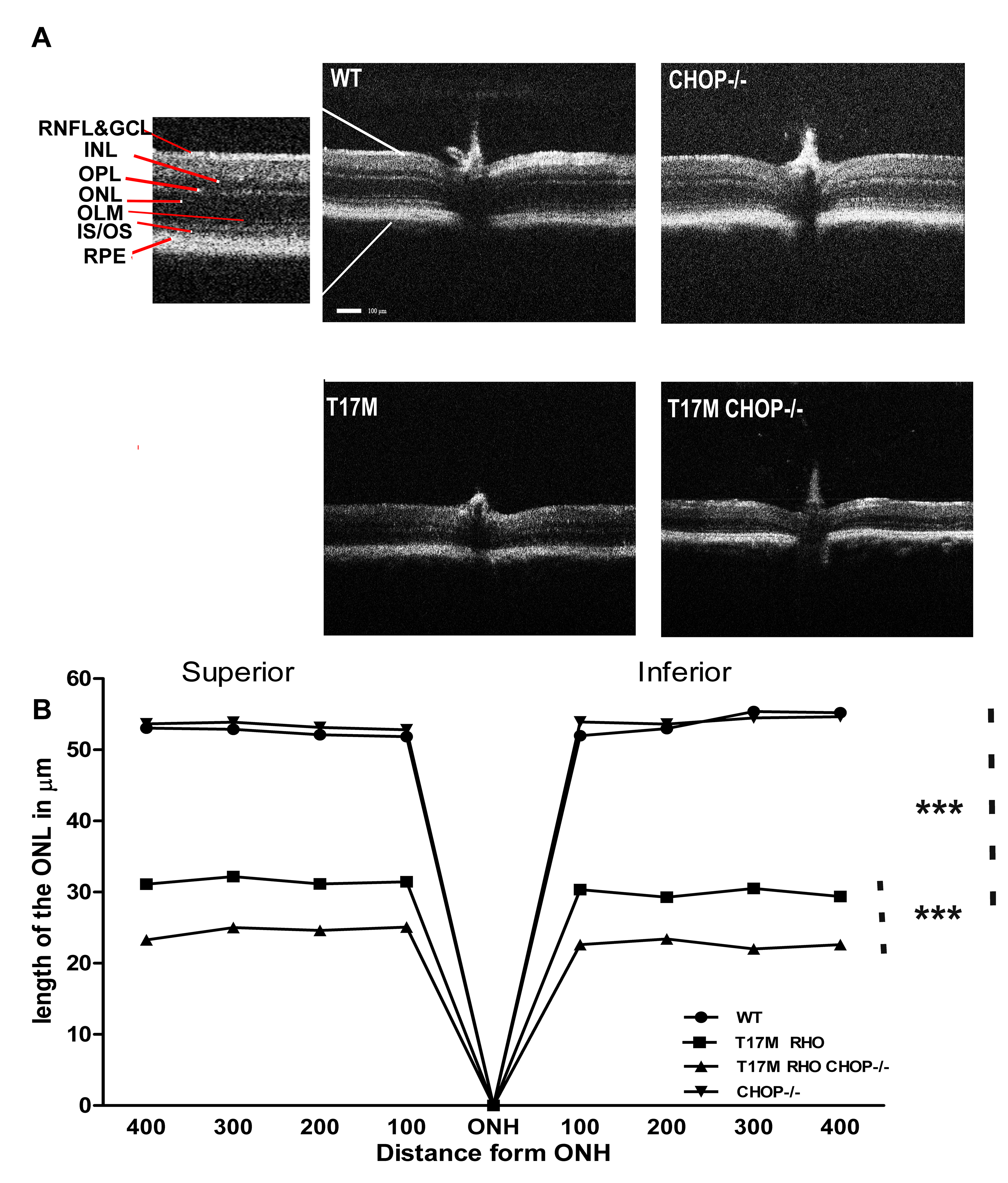

Supplement: Figure S2 — Alteration of retinal structure in the T17M RHO CHOP−/− retinas. A: SD-OCT images were captured in 1-month-old live mice. B: Thickness of the Outer Nuclear Layer (ONL) was measured in the superior and inferior retinal hemispheres in 1-month old mice. Measurements were made at a distance of 100, 200, 300 and 400 µm from the optic nerve head. The ONL in the T17M RHO and T17M RHO CHOP−/− retinas was significantly thinner compared to that of wild-type or CHOP−/− retinas at corresponding time points. Also, significant difference (*** P value <0.001) in ONL thickness was observed between the T17M RHO and T17M RHO CHOP−/− retinas. RNF&GCL: retinal nerve fiber and ganglion cell layer; INL: Inner nuclear layer, OPL: Outer plexiform layer, ONL: Outer nuclear layer, OLM: Outer limiting membrane, IS/OS; inner and outer segments of photoreceptors and RPE: retinal pigment epithelium. (TIF) [file pone.0063205.s002.tif]
